# SARS-CoV-2 in Zimbabwe: milestones and challenges faced towards achieving the expected 60% herd immunity

**DOI:** 10.11604/pamj.2021.39.255.30331

**Published:** 2021-08-20

**Authors:** Vinie Kouamou, Richard Matarise, Elizabeth Dos Santos, Nyasha Elose, Justen Manasa

**Affiliations:** 1 Faculty of Medicine and Health Sciences, University of Zimbabwe, Harare, Zimbabwe

**Keywords:** COVID-19, Vaccine, Zimbabwe

## Abstract

In response to COVID-19 pandemic, the Zimbabwe government put in place various rigorous measures to curb the spread of the virus. Although roll-out and access to COVID-19 vaccines in Africa have been slow, the World Health Organization (WHO)-led COVID-19 Vaccines Global Access (COVAX) consortium and the African vaccine acquisition task team are striving to provide 720 million doses of COVID-19 vaccines to achieve 60% coverage in Africa by June, 2022. In line with this, the Zimbabwe vaccination programme commenced on the 26^th^ February 2021 and as of 9^th^ June 2021, approximately, 2.6% of the population have been fully vaccinated in the country. Although the COVID-19 pandemic has crippled the economy and caused significant strain on the public health system, much has been done in the country since the first case was recorded (20^th^ March 2020). However, much more needs to be done to finally reach the expected 60% herd immunity by June 2022.

## Perspectives

**Introduction:** the severe acute respiratory syndrome coronavirus 2 (SARS-CoV-2) has since spread globally presenting one of the most serious worldwide health crises, with highly negative impacts on health systems and socio-economic costs in Zimbabwe. However, the Zimbabwean government has done incredibly well in scaling up the corona virus disease 2019 (COVID-19) vaccines and made it accessible to the population. The scale up of the vaccine programme together with the vigorous COVID-19 containment measures implemented in the country have helped in curbing the spread of the virus. However, the country has been experiencing a surge in cases and battling a deadly third wave. In this perspective, we: 1) firstly highlight the status of COVID-19 in Zimbabwe; 2) secondly, acknowledge all the remarkable milestones Zimbabwe has made in controlling the pandemic; 3) thirdly, share the challenges faced towards achieving the expected 60% herd immunity; 4) finally, present the issues that should be comprehensively and proactively addressed in the country to achieve this herd immunity and possibly end the COVID-19 pandemic.

**Severe acute respiratory syndrome coronavirus 2 (SARS-CoV-2) in Zimbabwe:** the Coronavirus disease of 2019, caused by SARS-CoV-2 was first identified in Wuhan, China late 2019 and spread across the globe in early 2020 [1]. The government of Zimbabwe recorded its first case on the 20^th^ March 2020 [2] and declared COVID-19 a national disaster on the 27^th^ March 2020. In response to the COVID-19 disease, the government put in place various significant measures including multiple national lockdowns to curb the spread of the virus. As of 31^st^ March 2021, the country has recorded 36,882 cases of which 34,686 recoveries and 1,523 deaths [3] and the national case fatality rate stands at 4.1%. Like many other African countries, the Zimbabwe government came across many challenges in response to COVID-19 pandemic. These challenges included access to laboratory facilities for SARS-CoV-2 testing and scaling up testing, which resulted in the low number of tests carried out on the population. Additionally, sequencing data for the detection of local SARS-CoV-2 variants circulating was non-existent until recently.

**Strides made by the Zimbabwean government in curbing the COVID-19 pandemic:** despite all these challenges, particularly socio-economic and public health pressures, Zimbabwe maintained vigorous containment measures (e.g. complete national lockdown for 21 days, maintaining social and physical distancing and mandatory wearing of facial masks in public places) against the spread of the virus. As of 9^th^ June 2021, Zimbabwe recorded the second lowest prevalence of cumulative SARS-CoV-2 cases (39,432) amongst all the other Southern African Development Community countries (Eswatini (18,632), Botswana (56,217), Namibia (57,577), Mozambique (71,019), Zambia (98,376) and South Africa (1,691,491)) [3]. Encouragingly, the country has made remarkable strides in: a) increasing SARS-CoV-2 testing by decentralizing the testing capacity throughout the country with both public and private facilities taking part; b) assessing the rate of SARS-CoV-2 seroprevalence (<5%) in the country (data not yet published); c) identifying the circulating SARS-CoV-2 variants in the country and; d) finally rolling-out COVID-19 vaccines (Sinopharm/Sinovax vaccines, Covaxin and Sputnik vaccines).

In January 2021, 95% of the SARS-CoV-2 samples sequenced in Zimbabwe had the D614G mutation on the spike protein which was associated with higher transmissibility than the ancestral strain [4]. By March 2021, surveillance of SARS-CoV-2 in Zimbabwe showed dominance of variants of concern. The identified variants of concern included the previously reported B.1.351 (501Y.V2) which was originally identified in South Africa [5] and was recently named the Beta variant by the World Health Organization (WHO). Strikingly, this Beta variant accounted for the majority (95%) of the sequenced cases. The other variants of concern included the A.23.1 and C.2 variants [6]. Furthermore, on the 20^th^ May 2021, the Zimbabwe government revealed that the B.1.617 variant (the Delta variant) predominantly from the republic of India was detected at the localized outbreak in one of the regions in Zimbabwe (Kwekwe).

Although roll-out and access to COVID-19 vaccines in Africa have been slow, the WHO reports that at least 49 countries are rolling out COVID-19 vaccines on the continent. Despite the fact that, more vaccine candidates were proven efficacious against SARS-CoV-2 and authorized for use, vaccine acceptance rates were low and hesitancy was high (at the beginning of the pandemic) in Zimbabwe. The low vaccine acceptance rate and high hesitancy were similarly observed in other African countries [7]. However, the Zimbabwe vaccination programme kicked off on the 26^th^ February 2021 and as of 9^th^ June 2021, a total of 1.66 million doses of vaccines have been received in the country, of which 1.08 million doses have been administered. Slightly over 689,000 people have received their first dose and 394,000 their second dose of Sinopharm/Sinovax vaccines [3]. This suggests that, approximately 2.6% (394,000/15,000,000 people) of the population have been fully vaccinated, representing the highest number in the Southern Africa region.

However, the scaling-up of the vaccine has to continue meticulously to administer another 9,000,000 doses, in order to achieve 60% coverage of the population by June, 2022 as per the initiative of the African vaccine acquisition task team of the African Union and the WHO-led COVAX consortium. Encouragingly, a remarkable decrease in the number of SARS-CoV-2 cases and total deaths have been observed in the country since the vaccination started until recently (8^th^ June 2021), compared to the rising levels before vaccination. This observation indeed cements the fact that, an increased rollout of vaccines could be the hope to control the COVID-19 pandemic and reboot the economy and society.

**Challenges faced towards achieving the expected 60% herd immunity:** although the administration of the vaccine is going on very well in Zimbabwe, many people are still not properly informed regarding the decentralization of the vaccination centers. Additionally, the administration of the vaccine in the country is still largely done on paper records and there is no adequate system to track people who are due for their second dose. Due to this lack of a proper tracking system, the country has run out of the second doses of the Sinopharm/Sinovac vaccines, leaving thousands of people partially vaccinated as of 9^th^ June 2021. Regrettably, Zimbabwe has been experiencing a surge in cases and have been battling the third wave since the 4^th^ June 2021 to date, attributed to a general complacency in adhering to the preventative measures both in the communities and work places [8]. Moreover, the opening of the tobacco sales floor markets and the gold mining business has led to people gathering. Thus, farming and mining towns have been greatly affected and classified as hotspots in the country.

**Issues to be addressed to achieve the 60% herd immunity:** the WHO-led COVAX consortium and the African vaccine acquisition task team are striving to provide 720 million doses of COVID-19 vaccines to achieve 60% coverage in Africa by June, 2022 [9]. However, the Zimbabwe government should strictly enforce preventive measures to curb this surge in cases and deaths and possible stop the third wave in the country. Additionally, the Zimbabwe medical community should play its part in educating and widely informing its population about the vaccines being administered so as to reduce vaccine hesitancy. Additionally, they should focus on local capacity for vaccine distribution and pharmacovigilance. Furthermore, post-vaccination surveys should be undertaken to understand the real efficacy of these vaccines in our population. Specifically, the efficacy or effectiveness of these vaccines against the Beta and Delta variants circulating in the country. These issues should be comprehensively and proactively addressed to achieve herd immunity, and end the COVID-19 pandemic in the country.

**Conclusion:** much has been done in the country since the SARS-CoV-2 pandemic started, however, much more needs to be done to reach the expected 60% herd immunity by June 2022.
